# Electroacupuncture for diminished ovarian reserve-associated infertility with a live birth: A case report

**DOI:** 10.1097/MD.0000000000042537

**Published:** 2026-03-06

**Authors:** Kaoling Wen, Ting Peng, Chunzhi Tang, Wei Yi

**Affiliations:** a Medical College of Acu-Moxi and Rehabilitation, Guangzhou University of Chinese Medicine, Guangzhou, China; b Chinese Medicine Guangdong Laboratory, Guangzhou University of Chinese Medicine, Zhuhai, China; c South China Research Center for Acupuncture and Moxibustion, Medical College of Acu-Moxi and Rehabilitation, Guangzhou University of Chinese Medicine, Guangzhou, Guangdong, China.

**Keywords:** case report, diminished ovarian reserve, electroacupuncture, infertility, live birth

## Abstract

**Introduction:**

Diminished ovarian reserve (DOR) refers to a decrease in the quantity and/or quality of oocytes in the ovaries, resulting in decreased fertility. DOR is one of the primary causes of infertility in women. Electroacupuncture therapy has been widely used in gynecological diseases. To our knowledge, there are currently no published case reports of electroacupuncture for two intermittent treatments of DOR-associated infertility. We present a case of electroacupuncture in two separate treatment sessions for DOR-associated infertility, resulting in a successful live birth following an initial miscarriage.

**Rationale:**

Infertility affects reproductive-aged couples and significantly impacts demographics trends. Electroacupuncture may be effective for DOR-associated infertility.

**Patient concerns:**

A 30-year-old woman presented with DOR-associated infertility and expressed concern about the substantial costs associated with assisted reproductive technology.

**Diagnoses:**

The patient was diagnosed with DOR-associated infertility and early threatened abortion. According to traditional Chinese medicine theory, the diagnosis was infertility (a deficiency in both the spleen qi and kidney qi, accompanied by blood stasis).

**Interventions:**

Based on the patient’s condition, we employed electroacupuncture to tonify qi and blood, and to invigorate blood circulation.

**Outcomes:**

After the first treatment, anti-müllerian hormone levels and antral follicle count increased, whereas baseline follicle-stimulating hormone levels decreased. The patient conceived but experienced a miscarriage due to embryonic chromosomal abnormalities despite normal parental karyotypes. Following the second treatment, the patient achieved a spontaneous pregnancy and delivered a healthy baby.

**Lessons:**

Electroacupuncture may improve pregnancy outcomes in DOR-associated infertility, and prolonging the treatment course could be beneficial for achieving a live birth.

## 1. Introduction

Diminished ovarian reserve (DOR), also known as ovarian insufficiency, refers to a decrease in the quantity and/or quality of oocytes in the ovaries. This decline leads to a reduction in ovarian reproductive and endocrine functions, thereby resulting in decreased fertility. It is characterized by lower levels of anti-müllerian hormone (AMH) and antral follicle counts (AFC), along with an increase in follicle-stimulating hormone (FSH). It is often associated with irregular menstruation, endocrine dysfunction, and poor responses to ovarian stimulation, which may ultimately progress to ovarian failure. DOR is one of the primary causes of infertility in women.^[1,2]^

For patients with DOR-associated infertility, assisted reproductive technology is the most commonly used therapy to facilitate conception. Throughout the treatment cycle, various controlled ovulation stimulation regimens and pretreatment medications are usually employed to increase the pregnancy rate. However, despite advancements in assisted reproductive technology, these interventions do not fully address the challenges associated with DOR, such as low oocyte quality, which can lead to aneuploid pregnancies and miscarriages,^[3–5]^ and many patients express concerns about the substantial costs associated with assisted reproductive technology.

Acupuncture is a traditional Chinese medical therapy that involves stimulating acupoints with stainless steel needles. It has been widely used in gynecological diseases, such as endometriosis,^[6]^ polycystic ovary syndrome,^[7]^ and premature ovarian failure.^[8]^ Here, we contribute to the existing literature by presenting a case of a patient who underwent electroacupuncture for DOR-associated infertility, achieving natural pregnancy twice and resulting in one live birth.

## 2. Case presentation

### 2.1. Clinical presentation

**Figure 1.**
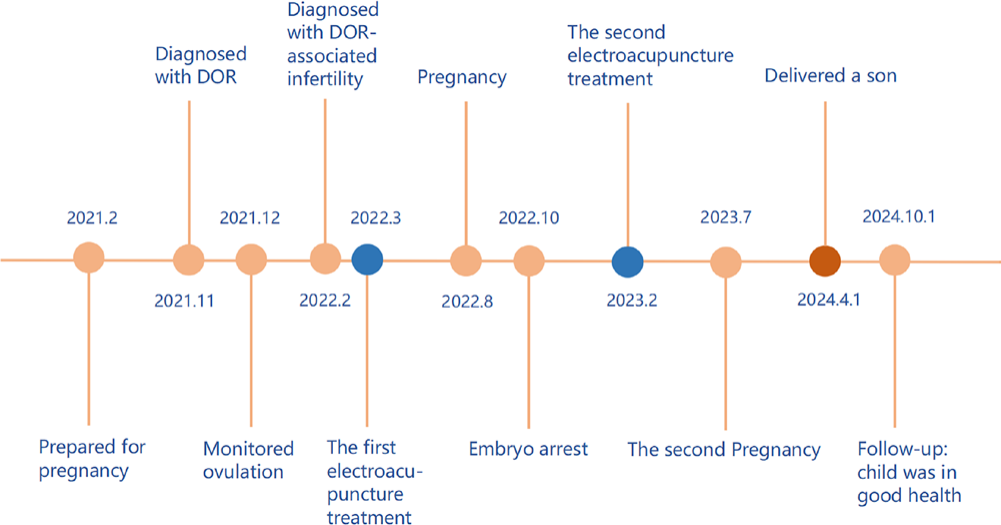
The timeline of the electroacupuncture for DOR-associated infertility. DOR = diminished ovarian reserve.

**Figure 2.**
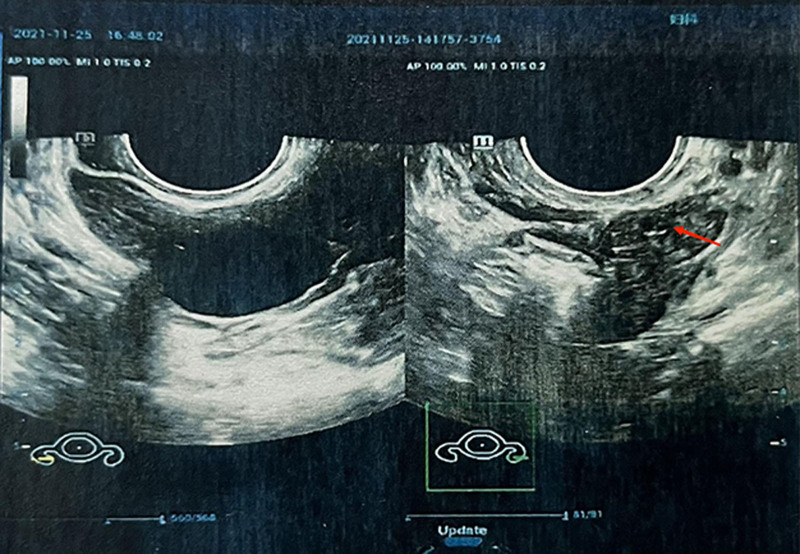
(November 2021) Transvaginal ultrasound showed 1 antral follicle count (AFC) observed in the left ovary.

**Figure 3.**
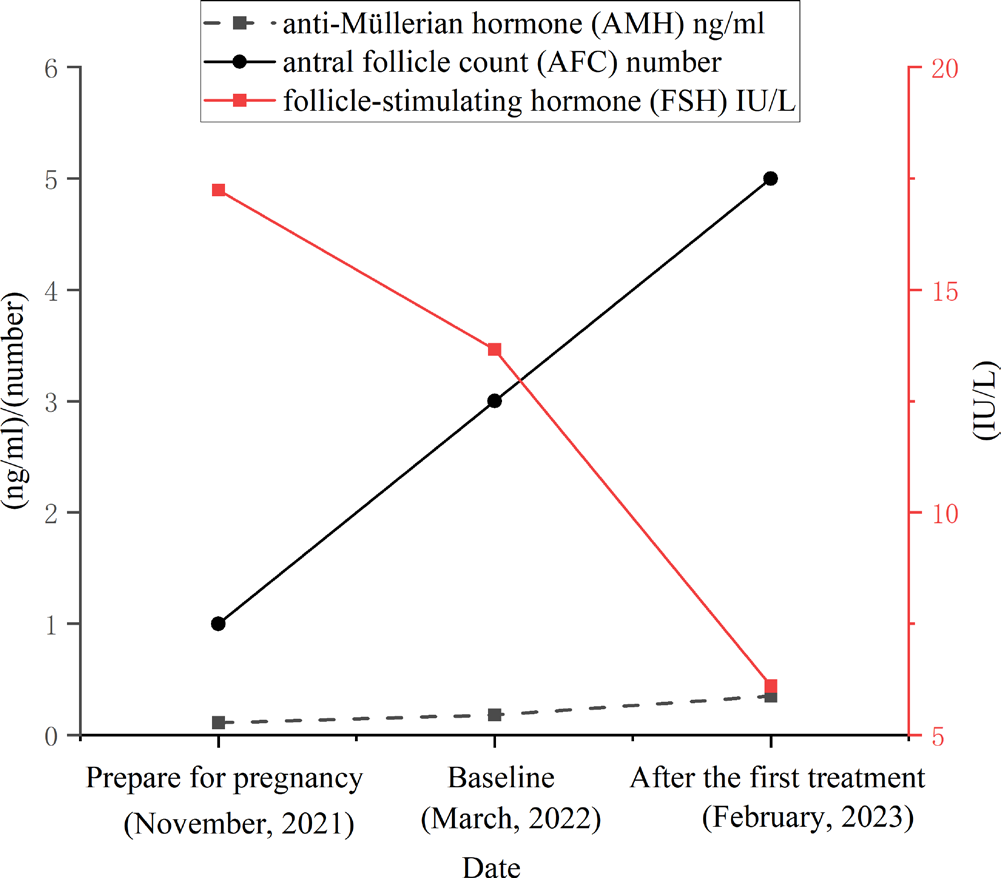
Change from baseline in AMH, FSH, and AFC. AFC = antral follicle count, AMH = anti-müllerian hormone, FSH = follicle-stimulating hormone.

A 29-year-old Chinese female presented to the Guangdong Maternal and Child Healthcare Centre on November 25, 2021 (see Fig. 1). Nine months prior, she had begun actively preparing for pregnancy. Her partner’s spermiograms and sperm morphology were normal according to the World Health Organization criteria; however, they had not achieved pregnancy. Her menstrual cycle was irregular, with an AMH level of 0.11 ng/mL, a FSH level of 17.24 IU/L on the third day of her menstrual cycle, and an AFC observed in the left ovary. (see Figs. 2 and 3). The patient was diagnosed with DOR by the physician, and then she began subsequently monitoring ovulation. In December 2021, a dominant follicle was observed in the right ovary, whereas no dominant follicles were detected in either ovary in January 2022. The patient did not achieve pregnancy until March 2022, indicating a year of unsuccessful conception attempts. She then presented to the acupuncture outpatient department at the First Affiliated Hospital of Guangzhou University of Traditional Chinese Medicine. During the last menstrual cycle before her visit, her AMH level was 0.179 ng/mL, with FSH measured at 13.66 IU/L on the third day of her menstrual period, LH at 7.87 IU/L, and E2 at 117.0 pmol/L. The patient’s last menstrual period occurred on February 19, 2022, lasting for 9 days. The cycle was irregular and accompanied by dysmenorrhea, with moderate flow, dark red color, and blood clots. She also experienced a poor appetite, disturbed sleep, sweating, and heightened sensitivity to stress.

### 2.2. The first electroacupuncture treatment

Guided by the fundamental theories of traditional Chinese medicine, the acupuncturist diagnosed the patient with deficiency of both spleen qi and kidney qi, accompanied by blood stasis. The selected acupuncture points target the root cause of the patient’s condition. The acupuncture points used include Baihui (GV20), Shenting (GV24), Zhongwan (CV12), Tianshu (ST25), Guilai (ST29), Qihai (CV6), Guanyuan (CV4), Xuehai (SP10), Zusanli (ST36), Sanyinjiao (SP6), Gongsun (SP4), Hegu (LI4), Yinlingquan (SP9), and Fuliu (KI7). All acupuncture points were approached bilaterally, except for GV20 and GV24. The locations of these points were determined according to the World Health Organization Standard Acupuncture Point Locations in the Western Pacific Region (see Table 1).

**Table 1:** The location and operation of acupoints.

Acupoint	Localization	Insertion depth (mm)	Needling angle (◦)
Baihui (GV20)	On the head, 5 B-cun superior to the ante rior hairline, on the anterior median line.	10–30	15 (horizontal)
Shenting (GV24)	On the head, 0.5 B-cun superior to the an terior hairline, on the anterior median line.	10–30	15 (horizontal)
Zhongwan(CV12)	On the upper abdomen, 4 B-cun superior to the center of the umbilicus, on the anterior median line.	10-30	90 (vertical)
Tianshu (ST25)	On the upper abdomen, 2 B-cun lateral to the center of the umbilicus.	10-30	90 (vertical)
Qihai (CV6)	In the lumbar region, at the same level as the inferior border of the spinous process of the third lumbar vertebra (L3), 1.5 B-cun lateral to the posterior median line	10-30	90 (vertical)
Guanyuan (CV4)	In the lumbar region, at the same level as the inferior border of the spinous process of the fifth lumbar vertebra (L5), 1.5 B-cun lateral to the posterior median line.	10-30	90 (vertical)
Xuehai(SP10)	On the anteromedial aspect of the thigh, on the bulge of the vastus medialis muscle, 2 B-cun superior to the medial end of the base of the patella.	10-30	90 (vertical)
Zusanli (ST36)	On the anterior aspect of the leg, on the line connecting ST35 with ST41, 3 B-cun inferior to ST35.	10-30	90 (vertical)
Sanyinjiao(SP6)	On the tibial aspect of the leg, posterior to the medial border of the tibia, 3 B-cun superior to the prominence of the medial malleolus.	10-30	90 (vertical)
Gongsun(SP4)	On the medial aspect of the foot, antero inferior to the base of the first metatarsal bone, at the border between the red and white flesh.	10-15	90 (vertical)
Hegu (LI4)	On the dorsum of the hand, radial to the midpoint of the second metacarpal bone.	10-20	90 (vertical)
Yinlingquan (SP9)	On the tibial aspect of the leg, in the de pression between the inferior border of the medial condyle of the tibia and the medial border of the tibia	10-30	90 (vertical)
Fuliu (K17)	On the posteromedial aspect of the leg, anterior to the calcaneal tendon, 2 B-cun superior to the prominence of the medial malleolus	10-30	90 (vertical)

First, the patient was placed in a supine position. After the routine disinfection of the points, sterile acupuncture needles (Suzhou Medical Appliance Factory, Suzhou, China) were inserted into the designed points. Needles measuring 0.20 mm × 25 mm were used for GV20 and GV24, while other points received needles measuring 0.25 mm × 40 mm. The acupuncturist then employed a reinforcing-reducing technique at the points until the deqi sensation was achieved. (characterized by feelings of soreness, numbness, distension, and heaviness). The needle handles of each side of ST25 and ST29 were connected to a pair of electrodes from an electroacupuncture therapy instrument (Suzhou Medical Appliance Factory, Suzhou, China) to receive electrical stimulation delivered in a dilatational wave at 2 Hz and a current of 1–2 mA. Each electroacupuncture treatment session lasted for 30 minutes. The patient received treatment once every 5 to 7 days for a total of six treatment courses, with four sessions constituting one course.

### 2.3. The first clinical outcome

**Figure 4.**
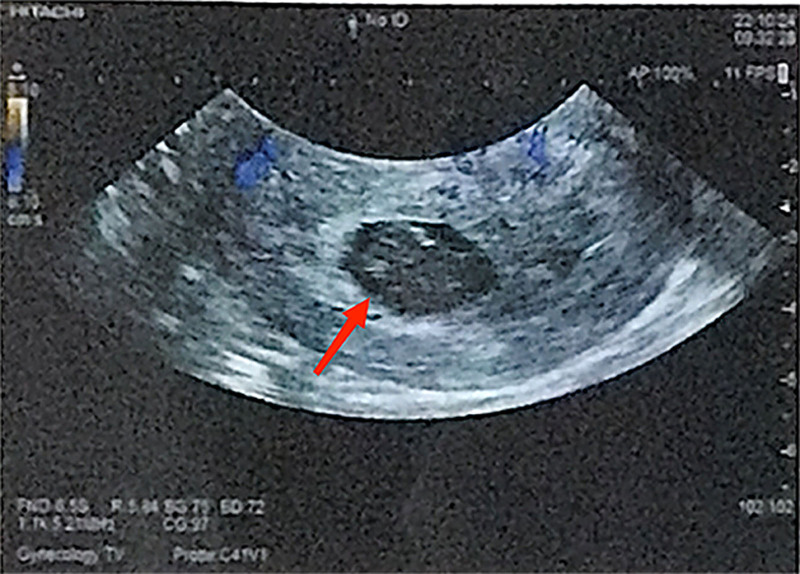
(October 2022) Transvaginal ultrasound showed an arrested fetus.

On September 5, 2022, the patient’s human chorionic gonadotropin (hCG) test returned positive. An ultrasound performed on September 28, 2022, confirming an intrauterine pregnancy at five weeks of gestation. On October 17, 2022, the ultrasound revealed an intrauterine pregnancy with a live fetus at 7 + weeks of gestation. However, an ultrasound on October 24, 2022, indicated an arrested fetus (see Fig. 4). The chromosomal examination revealed a sporadic chromosomal abnormality in the fetus, with both parents exhibiting normal chromosome profiles.

### 2.4. The second electroacupuncture treatment

On February 15, 2023, the patient returned to the hospital for a reexamination. The hormone levels from the last menstrual cycle were as follows: AMH 0.35 ng/mL, FSH 6.10 IU/L, LH 1.71 IU/L, and E2 153 pmol/L. The ultrasound revealed an AFC of 5. The last menstrual period began on January 22, 2023 and lasted for 8 days. The patient reported no dysmenorrhea, no blood clots, no back pain, and no breast swelling. Then the patient received electroacupuncture treatment once every 5 to 7 days for a total of four treatment courses, with four sessions constituting one course. The patient had good compliance.

### 2.5. The second clinical outcome

**Figure 5.**
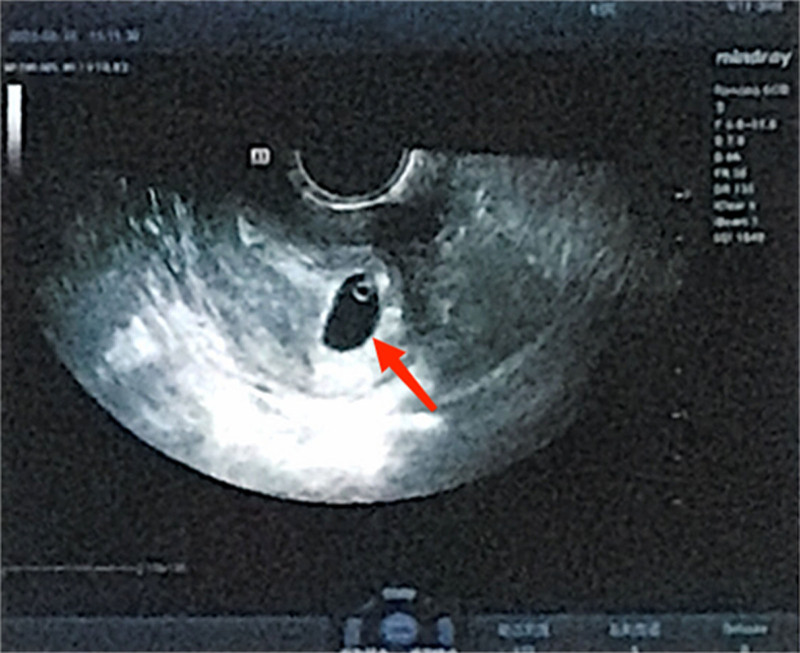
(August 2023) Transvaginal ultrasound showed a pulsating fetal heartbeat, revealed an intrauterine pregnancy with a live fetus at approximately 6 weeks gestation.

After four sessions of acupuncture treatment, the patient tested positive for hCG on July 30, 2023. A transvaginal ultrasound performed on August 16, 2023, revealing an intrauterine pregnancy with a live fetus at approximately 6 weeks gestation, evidenced by the presence of a pulsating fetal heartbeat (see Fig. 5).

### 2.6. Follow-up

**Figure 6.**
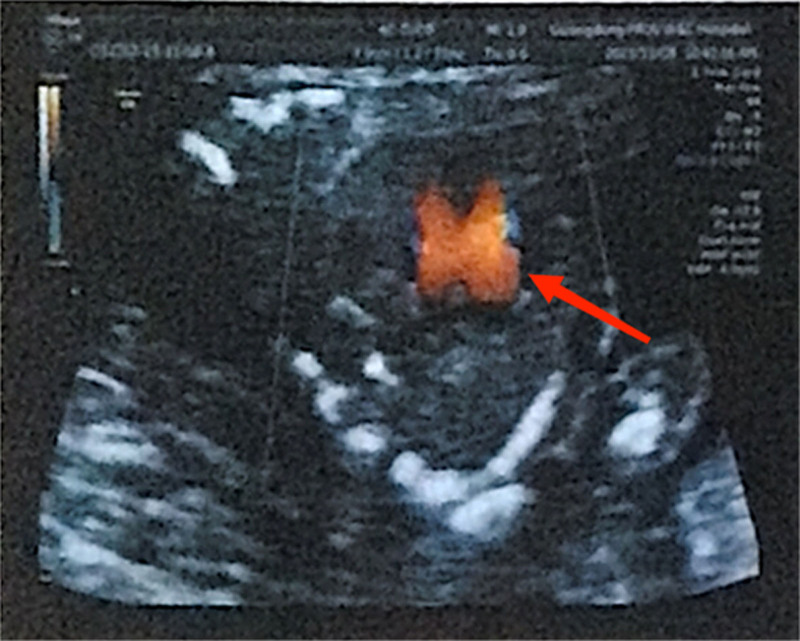
(November 2023) Ultrasound showed an intrauterine pregnancy with a single live birth.

The first follow-up visit with the patient was conducted three months after the treatment. An ultrasound revealed an intrauterine pregnancy with a single live birth (see Fig. 6). The prenatal genetic testing indicated no abnormalities in sex chromosomes or autosomal numbers, and there was no deletion of exons 7 and 8 of the SMN1 gene. Follow-up visits were scheduled at six-month intervals. During the second follow-up in May 2024, the patient reported the delivery of a son via forceps on April 1, 2024. She had a good appetite but experienced symptoms of hypersomnia. The baby was healthy. At the third follow-up in October 2024, both the mother and the child were in good health.

## 3. Discussion

Infertility is estimated to affect between 8% to 12% of reproductive-aged couples worldwide,^[9]^ and has a significant impact on demographics. Infertility caused by DOR accounts for approximately 10% of female infertility cases. Although therapeutic management of DOR remains debated,^[10]^ assisted reproductive technology remains the most commonly used treatment. However, oocyte quality significantly affects pregnancy success rates.^[11]^ In this case of DOR-associated infertility, we selected clinical pregnancy rate and LBP as primary outcomes, and AMH, baseline FSH, and AFC as secondary outcomes, all of which are recommended for assessing DOR-related infertility. Twenty-four sessions of electroacupuncture treatment over 24 weeks resulted in the patient’s spontaneous pregnancy, an increase in AMH and AFC levels, and a decrease in baseline FSH levels. However, there was a miscarriage due to chromosomal abnormalities despite both parents being chromosomally normal. As is know, DOR can lead to spontaneous abortion and chromosomal aberrations due to decreased oocyte quality. Subsequently, after 16 sessions of treatment over 16 weeks, the patient achieved a second natural pregnancy, resulting in a successful delivery of a baby boy. Interestingly, the patient had experienced one year of infertility, and was diagnosed with DOR-associated infertility. The spontaneous pregnancies and one live birth within two years appear to be associated with the receipt of electroacupuncture.

The observations made in this case are consistent with findings from two studies. A meta-analysis, performed by Xu et al. (2023), which included 25 randomized controlled trials with a total of 4757 patients, demonstrated that compared with blank control and placebo acupuncture, acupuncture administered during embryo transfer improved both the clinical pregnancy rate (43.6% vs. 33.2%, *P *< .00001) and live birth rate (38.0% vs. 28.7%, *P *< .00001) in women undergoing in vitro fertilization.^[12]^ Additionally, a systematic review published in 2023 encompassing 312 original randomized controlled trials with 65,388 participants indicated that acupuncture, compared to sham acupuncture, no adjunctive treatment, western or Chinese medicine treatment, was beneficial in increasing the clinical pregnancy rate (RR = 1.31; 95% CI: 1.13-1.52; *P* = .0004; *I*^2^ = 66%). However, no statistically significant difference was found in live birth rate outcomes.^[13]^ These discrepancies may stem from variations in study selection criteria, as well as differences in the definitions of participants, interventions, and outcomes among reviewers.

Electroacupuncture, as a novel therapy for DOR, has been demonstrated to modulate diverse cellular functions and mechanisms. Electroacupuncture can inhibit the elevation of FSH and LH levels^[14]^ and enhance the utilization efficiency of E2 by targeting the PI3K/AKT/mTOR signaling pathway,^[15]^ thus restoring the depressed function of the hypothalamic–pituitary–ovarian axis.^[16]^ Similarly, there are studies have shown that electroacupuncture reduces the levels of FSH and LH and increases the level of E2 in rats with cyclophosphamide-induced POF by inhibiting the expression of p38MAPK protein in ovarian tissues.^[17]^ Our findings appear consistent with these observations. Another study^[18]^ revealed that electroacupuncture treatment can reduce the oxidative stress damage mediated by DOR through inhibiting the expression of bta-miR-7857-3p_R-1, mdo-miR-26b-5p_R + 1_1ss10TC and rno-miR-92b-3p in ovarian tissues, thereby improving ovarian function. Additionally, some studies have found that acupuncture intervention inhibits the early and late apoptosis of ovarian granulosa cells in PCOS rats by targeting miR-21-3p via LncMEG3.^[19]^ Electroacupuncture promotes the proliferation of granulosa cells through the PI3K-Akt signaling pathway, facilitating follicular development and negative feedback regulation of sex hormones.^[20]^ Moreover, electroacupuncture can alter the gut microbiota, effectively inhibit ovarian oxidative stress and the accumulation of Fe^2+^ in mice with premature ovarian failure,^[21]^ thus increasing the number of mature follicles and improving the levels of sex hormones, which is consistent with the findings of Zhang et al.^[22]^

The strength of this report lies in its status as the first to describe the effects of electroacupuncture on women with DOR-associated infertility, focusing on primary outcomes of clinical pregnancy and live births. However, this study is limited by its reliance on a single case, which may restrict the generalizability of the findings to the broader population of women with DOR-associated infertility. The absence of a control group makes it difficult to draw firm conclusions about the effectiveness of electroacupuncture. Additionally, the lack of a blinding protocol for the acupuncture procedures may have introduced bias into the results. Furthermore, the exploration of the minimum treatment duration and frequency of effective electroacupuncture treatment for DOR-associated infertility may be needed.

## 4. Conclusion

This article reports a case of electroacupuncture in two separate treatment sessions for DOR-associated infertility, which subsequently resulted in a successful live birth. Our report suggests that alternative therapies such as electroacupuncture may promote pregnancy, and extending the treatment course may improve live birth rates. However, further randomized controlled studies with larger sample sizes are still needed to validate the efficacy in this context.

## Author contributions

**Investigation:** Kaoling Wen, Ting Peng.

**Resources:** Ting Peng.

**Supervision:** Wei Yi.

**Validation:** Wei Yi.

**Writing – original draft:** Kaoling Wen, Ting Peng.

**Writing – review & editing:** Kaoling Wen, Chunzhi Tang, Wei Yi.
